# Trends in Medical Expenditures Prior to Diabetes Diagnosis: The Early Burden of Diabetes

**DOI:** 10.1089/pop.2019.0143

**Published:** 2021-02-02

**Authors:** Tamkeen Khan, Jianing Yang, Gregory Wozniak

**Affiliations:** Improving Health Outcomes, American Medical Association, Chicago, Illinois, USA.

**Keywords:** diabetes, prediabetes, medical expenditures

## Abstract

Diabetes is a costly chronic condition in the United States. The incremental increase in costs of the disease can begin and accelerate prior to first diagnosis. This study conducts a retrospective analysis of claims data from Truven Health MarketScan^®^ Commercial Claims Database to track per capita annual medical expenditures among a single panel of commercially insured patients for 5 years preceding a new diabetes diagnosis. Case subjects, defined as individuals newly diagnosed with diabetes in 2014, are compared to control subjects, defined as individuals who do not have a diabetes diagnosis. Arithmetic means, compound annual growth rates, and propensity score matching models are used to track the differential in expenditures across health care sectors. This analysis finds that the incremental rise in costs of diabetes are shown to begin at least 5 years before diagnosis of the disease and accelerate immediately after diagnosis. Results of the matching model suggest that the newly diagnosed case subjects spent $8941 more than control subjects not diagnosed with diabetes over the span of 5 years, with approximately $4828 in the year of diagnosis. The compounded annual growth rate in per capita annual medical expenditures between 2010–2014 was almost 9% higher among case subjects at 14.3% in the matched models. Results show that the rise in medical spending associated with diabetes begins well in advance of the first diabetes diagnosis and support the need to encourage physicians to implement timely identification and prevention efforts to reduce the economic burden of the disease.

## Introduction

The health care cost of diabetes in the United States in 2017 was estimated to be $237 billion, with another $90 billion in reduced productivity.^1^ Individuals with diagnosed diabetes incur average medical expenditures approximately 2.3 times higher than those without diabetes at roughly $16,750 per year, almost $9600 of which is because of the disease. Between 2012–2017, the economic costs of diabetes increased 26%, attributed to both the rise in prevalence of diabetes and the increased costs per person with diabetes.^1,2^ People with diabetes are more likely to have hypertension, to be hospitalized for a heart attack or stroke, and to die from a heart attack or stroke compared to their counterparts.^3,4^

Type 2 diabetes is often preceded by prediabetes, characterized by blood glucose levels that are elevated but not high enough to be diagnosed as diabetes.^3^ Individuals with prediabetes may exhibit poorer health and incur increased medical expenditures compared to those without prediabetes because of greater use of health care services, medications, and other health care products.^5^ Annual per capita health care spending was roughly $2700 more for those who had transitioned from prediabetes to diabetes among a commercially insured adult popuation.^6^

The economic costs of diabetes can be mitigated by participation in lifestyle change programs (LCPs), which are shown to successfully prevent or delay the onset of diabetes among their participants, such as those offered through the Centers for Disease Control and Prevention (CDC)-led National Diabetes Prevention Program.^7^ These programs are modeled after the original Diabetes Prevention Program research study and several subsequent translational research studies that suggest that a 5%–7% body weight loss reduced the incidence of diabetes by 58% with an average follow-up of 2.8 years.^8,9^

Prior research finds primary care consultations, drug utilization, and the incremental costs of diabetes begin in the years before diagnosis and grow at an accelerating rate approaching and immediately after diagnosis.^10–12^ This study builds on the existing literature by tracking patterns and trends in medical care for a single panel of commercially insured individuals all diagnosed with diabetes in the same year. Furthermore, this work estimates the rate of growth in spending over the 5 years for both patients newly diagnosed with diabetes and patients with no diagnosis of diabetes. The research team believes this work shows the importance of understanding early identification of prediabetes and the value of preventive interventions including referral and participation in CDC-recognized LCPs.

### Study data

This study utilized retrospective de-identified claims data from Truven Health MarketScan^®^ Commercial Claims Database from 2009–2014. These data integrate claims and enrollment information submitted to Truven under business agreements with large employers and commercial insurance carriers that provide private health care coverage for employees, their spouses, and dependents, as well as health insurance claims across the continuum of outpatient, inpatient, and pharmaceutical sectors. This database comprises a variety of fee-for-service, preferred provider organization, and capitated health plans. Dollar values are raw estimates and include patient premiums, co-payments, deductibles, and payments made by the insurance provider.

A total of 5 medical and surgical data files were merged for this study: claims data for outpatient services, inpatient admissions, inpatient services, outpatient pharmaceutical drugs, and enrollment data. Outpatient services data contain encounters and claims for services rendered in a doctor's office, hospital outpatient facility, emergency room, or other outpatient facilities. Inpatient admissions data contain encounters and claims associated with an admission (eg, hospital, physician, surgeon, independent laboratory claims) and met the criteria of a room and board claim present. Inpatient services data contain individual facility and professional services encountered during the inpatient admission.

Variables extracted from both the outpatient and inpatient files include age, payment, principal diagnosis code, 3 additional diagnosis codes, metropolitan statistical area (MSA), region, sex, employee classification, employment status, and the Market Scan national weight link. Outpatient pharmaceutical drug claims data were available for a large portion of the individuals in the medical/surgical and populations tables. Each record represents either a mail order or card program prescription drug claim. Finally, annual enrollment data contain a single record per person, per year data on indicators of enrollment and plan type in each month during the year. All data sets were merged by enrollee identification number.

Inclusion criteria for this analysis were continuously enrolled adults from 2010–2014, between the ages of 18–64 years, with no prior diagnosis of diabetes or other conditions associated with diabetes for at least 6 months. Pregnant women were excluded from this analysis. Diabetes claims were defined as *International Classification of Diseases, Ninth Revision* (ICD-9) codes for primary and secondary diagnosis of diabetes mellitus (all codes with prefix of 250 and 249) and other conditions associated with diabetes (357.2, all codes with prefix 362, 366.41, and all codes with prefix of 648).

Medical expenditures comprised payments made by insurance providers and individuals and were adjusted to constant 2010 US dollars using the average annual percent change in the Consumer Price Index for all urban consumers (CPI-U) for medical care.^13^ Cases with negative total annual expenditures were omitted from the analyses. Expenditures in each of the 3 categories of medical care services, as well as a combination of the totals for these sectors, were analyzed. Subjects were separated into 2 cohorts: case subjects, who were individuals newly diagnosed with diabetes in 2014, and control subjects, who had no documented diagnosis of diabetes by 2014.

## Methods

**FIG. 1.**
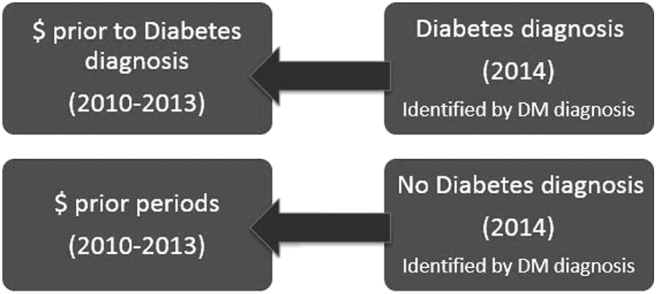
Study population. DM, diabetes mellitus.

This study retrospectively tracks trends in per capita annual medical expenditures for 5 years among a single panel of case and control subjects as shown in Figure 1. To compare variations in spending, the differences in the arithmetic means were calculated for all 3 sectors of health care between the 2 groups. Compound annual growth rates (CAGRs) in per capita expenditures were calculated between 2010–2014 to best track the geometric progression ratio and estimate the rate of growth in spending over 5 years.

Ideally, the preferred approach would be to compare spending for a person newly diagnosed with diabetes to themselves had they not developed diabetes, such that all the differences would be attributable to the disease. Therefore, a propensity score matching method was implemented to compare case subjects to control subjects. The model takes the form of P(X) = Pr (D = 1|X), where D = 1 indicates diabetes is diagnosed in 2014 and matches on the probability of diagnosis instead of attempting to create a match for each participant with the same value of X. This model aims to account for unobservables and obtain an unbiased and accurate measure of costs attributed to diabetes diagnosis. Matched control subjects who were not diagnosed with diabetes must be similar to the case subjects who were diagnosed with diabetes in 2014 so that the only difference is attributed to the disease.

The matching model in this analysis controlled for the following: patient age, sex, MSA, region, employee classification, and employment status (in 2014); and hypertension, chronic obstructive pulmonary disease, congestive heart failure, cancer diagnoses (includes ICD-9 codes for malignant neoplasm: 140–209.36), and metformin use (Common Procedural Terminology codes 1–27) in all years of the data.

Based on a univariate chi-square test of independence between characteristics and 2-sample *t* test, statistical significance was defined as a *P* value <0.05. All analyses were conducted using SAS 9.4 (SAS Institute Inc., Cary, NC) and STATA 14 (StatCorp LP, College Station, TX).

## Results

Descriptive statistics for the full sample (n = 683,680), the 17,207 case subjects diagnosed with diabetes in 2014, and the 532,175 control subjects without diabetes in 2014 are presented in Table 1. Among the full sample, most of the adults are between the ages of 45–64 (67%) with fewer individuals distributed in the younger age brackets. Additionally, sample distribution by sex shows a slightly higher proportion of females. The sample was heavily concentrated in the south and northeast geographic regions. Among employee classifications, slightly more than one quarter (26%) were salaried and more than one quarter (27%) were hourly.

**Table 1.** Descriptive Statistics for Sample

	Full sample	Case subjects	Control subjects	χ^2^
	n (%)	n (%)	n (%)	P value
**Total sample**	683,680 (100)	17,207 (100)	532,175 (100)	
Age^*^				<0.001
18–34 years	60,264 (9)	696 (4)	56,176 (11)	
35–44 years	121,891 (18)	2256 (13)	105,999 (20)	
45–54 years	200,834 (29)	5259 (31)	159,031 (30)	
55–64 years	256,670 (38)	8779 (51)	170,717 (32)	
Missing	44,021 (6)	217 (1)	40,252 (7)	
**Sex**				<0.001
Male	271,132 (40)	7451 (43)	202,178 (38)	
Female	412,548 (60)	9756 (57)	330,043 (62)	
Region^*^				<0.001
Northeast	147,737 (22)	4482 (26)	110,918 (21)	
Midwest	119,970 (18)	3186 (19)	90,790 (17)	
South	310,005 (45)	7865 (46)	240,578 (45)	
West	61,785 (9)	1454 (8)	49,560 (9)	
Unknown/missing	44,183 (6)	220 (1)	40,375 (8)	
Employee classification^*^				<0.001
Salary nonunion	111,256 (16)	2760 (16)	89,398 (17)	
Salary union	41,925 (6)	929 (5)	34,691 (7)	
Salary other	24,268 (4)	502 (3)	20,208 (4)	
Hourly nonunion	25,670 (4)	712 (4)	19,932 (4)	
Hourly union	94,472 (14)	3471 (20)	63,340 (12)	
Hourly other	63,803 (9)	1565 (9)	50,909 (10)	
Nonunion	12,638 (2)	375 (2)	10,402 (2)	
Union	2210 (<1)	55 (<1)	1693 (<1)	
Unknown	263,417 (39)	6621 (38)	201,394 (38)	
Missing	44,021 (6)	217 (1)	40,254 (8)	
Employee status^*^				<0.001
Active full-time	285,668 (42)	7179 (42)	230,791 (43)	
Active part-time or seasonal	10,374 (2)	249 (1)	8261 (2)	
Early retiree	65,004 (10)	2360 (14)	42,078 (8)	
Medicare eligible retiree	10,463 (2)	368 (2)	6375 (1)	
Retiree (status unknown)	112(<1)	3 (<1)	69 (<1)	
COBRA continuee	2249 (<1)	79 (<1)	1666 (<1)	
Long-term disability	840 (<1)	37 (<1)	549 (<1)	
Surviving spouse/dependent	2598 (<1)	109 (<1)	1534 (<1)	
Other/unknown	262,331 (38)	6607 (38)	200,644 (38)	
Missing	44,021 (6)	217 (1)	40,254 (8)	

*P* values for univariate chi-square test of independence between characteristics of control and case.

The full sample includes individuals who may have been diagnosed with diabetes 2010–2013.

All values with negative expenditures are excluded.

^*^ 2014 data.

COBRA, Consolidated Omnibus Budget Reconciliation Act.

Unfortunately, about 42% reported unknown or had missing data among the classification variable. Similarly, 44% reported active employment status, but another 44% reported unknown or missing status. Sixteen percent of the sample was salaried nonunion while 39% report unknown status. Forty-two percent report employee status as active full-time with another 38% being categorized as other/unknown. Overall, the proportions for the demographic data are similar among the full sample and the breakouts between case and control subjects. Univariate chi-square tests showed that all the demographic characteristics were associated with onset of diabetes (*P* < 0.001)

Table 2 shows the results of the propensity score matching model used to match a control subject who was not diagnosed with diabetes to a case subject based on the covariates used in the model. There is a differential in spending between the 2 groups, but the magnitude of the difference is truncated with the matching method. Per capita annual medical expenditures in constant 2010 dollars by expenditure category and year for the case subjects and the control subjects are displayed for both the unmatched and matched analysis. The expenditure magnitudes and trends for this sample are similar to those reported by Truven Health Analytics for those with employer-sponsored insurance and other research on privately insured individuals.^12,14,15^ The slight variations may be because of the exclusion criteria applications including age, pregnancy status, and negative dollar amounts for claims.

**Table 2.** Average Annual Per Capita Medical Expenditures by Sector and Year for Propensity Score Matched Models

	Unmatched			Matched			
	Case subjects ($)	Control subjects ($)	Difference ($)	Case subjects ($)	Control subjects ($)	Difference ($)	t Test* P *value
Outpatient services							
2014	6731	3994	2737	6731	4446	2285	<0.001
2013	5082	3811	1271	5082	4261	882	<0.001
2012	4726	3639	1087	4726	4026	700	<0.001
2011	4365	3397	968	4365	3874	491	<0.001
2010	4005	3245	760	4005	3775	230	0.046
Inpatient admissions and services							
2014	3628	1250	2378	3628	1554	2073	<0.001
2013	1915	1134	781	1915	1480	435	0.002
2012	1670	1008	662	1670	1364	306	0.047
2011	1442	947	495	1442	1353	88	0.417
2010	1166	881	285	1165	1217	−52	0.578
Pharmaceutical drugs							
2014	2804	1932	872	2804	2334	470	<0.001
2013	1800	1294	506	1800	1464	336	<0.001
2012	1708	1247	461	1708	1435	272	<0.001
2011	1626	1203	423	1626	1377	250	<0.001
2010	1571	1141	429	1571	1335	236	<0.001
Total							
2014	13,162	7175	5987	13,162	8334	4828	<0.001
2013	8799	6239	2559	8799	7204	1594	<0.001
2012	8103	5893	2210	8103	6826	1277	<0.001
2011	7432	5548	1884	7432	6604	828	<0.001
2010	6741	5267	1474	6741	6327	414	0.016

*P* values for 2-sample *t* tests for difference in the means between case and control subjects for the matched sample.

N = 13,896 case subjects, N = 387,371 control subjects.

All values are adjusted to constant 2010 dollars.

All observations with negative expenditures and missing data for matching models are excluded.

Results shown in Table 2 confirm there is a statistically significant spending differential between the 2 groups in all categories of the matched sample, with the exception of inpatient admissions and services in 2010 and 2011 (*P* < 0.05), as well as in the unmatched model (results not shown). Comparisons of the declines in mean bias from 23.6 for the unmatched sample to 1.8 for the matched sample, coupled with a high percent reduction in bias for all covariates (results not shown), indicate the propensity score matching model was successful at reducing bias between the 2 groups. In 2014, per capita outpatient spending was almost $2300 higher, pharmaceutical expenses were slightly less than $500 higher, and inpatient expenses were just over $2000 higher. Total expenses were nearly $5000 more for case subjects diagnosed with diabetes in 2014 compared to control subjects. As in the unmatched models, the case versus control spending differentials gradually increase from 2010 to 2014, becoming substantially widest in the year of diagnosis.

Overall, total per capita annual spending for outpatient, inpatient, and pharmaceuticals among case subjects rose 2.0 times from 2010 leading up to the diagnosis of diabetes in 2014, while spending rose 1.3 times for the control subjects. There is a statistically significant difference in mean expenditures between the 2 groups across all years and categories (*P* < 0.001), which gradually widens over time and is greatest in the year in which the individual is first diagnosed with diabetes.

Breakouts of CAGR by health care sector are reported in Table 3. Total CAGR in per capita annual expenditure more than doubles from the control to case subjects. This rate is highest for the inpatient setting among the newly diagnosed case subjects at almost 25.5%, which is nearly 5 times higher than for control subjects. Similarly, CAGR for outpatient services is more than 3 times higher for cases compared to control subjects. However, in the pharmaceutical sector, CAGR was similar for both cohorts at approximately 12%. Overall, increases in CAGR from the control to case subjects were larger in the matched cases versus unmatched cases; total CAGR was 8.6% higher over the 5-year period.

**Table 3.** Compound Annual Growth Rate Between 2010–2014 in Per Capita Annual Medical Expenditures, by Sector for Propensity Score Matched Models

	Unmatched		Matched	
	Case subjects (%)	Control subjects (%)	Case subjects (%)	Control subjects (%)
Outpatient services	10.9	4.2	10.9	3.3
Inpatient admissions and services	25.5	7.3	25.5	5.0
Pharmaceutical drugs	12.3	11.1	12.3	11.8
Total	14.3	6.4	14.3	5.7

## Discussion

This retrospective study among a single panel of commercially insured patients found that incremental costs of diabetes are shown to begin at least 5 years before diagnosis of the disease and to accelerate immediately after diagnosis. The matching model found that commercially insured individuals with newly diagnosed diabetes in 2014 spent $8941 more than those not diagnosed with diabetes from 2010–2014. This cost differential or steepest jump, approximately $4828, was greatest in 2014 when all patients were diagnosed with diabetes, consistent with other research in this area using a diabetes index date suggesting higher costs of health care related to initial diagnosis.^10,12^

Variation between CAGR for those diagnosed with diabetes was 20.5% higher for inpatient services, 7.6% higher for outpatient services, and only 0.5% higher for pharmaceutical drugs, yielding an 8.6% differential for total medical expenditures. Prior research shows that the majority of these costs are for conditions normally associated with diabetes or its complications, but it also shows prediabetes is associated with higher use of health care services, medication, and other health care products.^6,10,16,17^ Results from this study confirm that there is clearly higher medical care utilization and expenditures that begin to increase even prior to diabetes diagnosis.

Comparing average trends in expenditures of patients newly diagnosed with diabetes to those without diabetes provides a better understanding of the economic implications that could be associated with diabetes prevention efforts. Health care providers can utilize the US Preventative Services Task Force recommendations for screening for abnormal glucose and type 2 diabetes among adults aged 40 to 70 years who are overweight or obese.^18^ This would aid in early identification of individuals at risk for developing diabetes and allow for prompt treatment such as referral to a CDC-recognized LCP.

Results from this work coupled with findings from prior research^8^ suggest that early identification and action for high-risk patient populations may have both health and economic benefits. Assuming individuals with prediabetes are able to maintain their health and prevent progression to diabetes, these individuals would expect to see a positive net savings and return on investment in health care expenditures over time.^6,9^ Further, it is important to note that this net savings estimate of lifestyle interventions does not include the potential benefits from avoiding lost wages resulting from reduced productivity of the employed population.^19^

### Limitations

This study has certain limitations. First, constant 2010 dollar values are calculated using the medical care CPI-U. The individual components of medical care price index, however, are slightly misaligned to the expenditure categories captured in the claims data. Additionally, the data are not weighted to control for various sampling issues such as regional characteristics, given that 45% of the sample is drawn from the south and only 9% is drawn from the west. Finally, matching models may not work well when important unobservable differences between individuals diagnosed with diabetes and those not diagnosed exist. Given the limitations of claims data it was not possible to control for lifestyle choices and genetics, which are correlated with the chronic disease.

## Conclusion

The rise in medical spending associated with diabetes begins well in advance of the diabetes diagnosis. These expenditures rise drastically once diabetes is diagnosed. These results prompt the importance of encouraging physicians to identify individuals with prediabetes early and support preventive efforts such as the CDC-recognized LCPs to reduce the incidence and economic costs associated with diabetes.
